# Paracentral Bitemporal Hemianopsia and Acquired Supranuclear Ocular Motor Paresis Following Cardiac Surgery

**DOI:** 10.7759/cureus.44108

**Published:** 2023-08-25

**Authors:** Calvin W Wong, Ahmad Kharsa, Noor Adnan Rashid Laylani, Pamela Davila Siliezar, Andrew G Lee

**Affiliations:** 1 McGovern Medical School, University of Texas Health Science Center at Houston, Houston, USA; 2 John Sealy School of Medicine, University of Texas Medical Branch, Houston, USA; 3 Department of Ophthalmology, Houston Methodist Hospital, Houston, USA; 4 Departments of Ophthalmology, Neurology, and Neurosurgery, Weill Medical College of Cornell University, New York, USA; 5 Department of Ophthalmology, University of Texas Medical Branch, Galveston, USA; 6 Department of Ophthalmology, University of Texas MD Anderson Cancer Center, Houston, USA; 7 Texas A&M School of Medicine, Texas A&M University, Bryan, USA; 8 Department of Ophthalmology, University of Iowa Hospitals and Clinics, Iowa City, USA

**Keywords:** ophthalmoplegia, chiasmal stroke, lateral gaze palsy, gaze palsy, asomp

## Abstract

Acquired supranuclear ocular motor paresis (ASOMP) is a rare complication following cardiac surgery, characterized by limited voluntary eye motility. The exact cause and development of ASOMP after cardiac surgery remain unclear. We report a case of ASOMP with paracentral bitemporal hemianopsia with optic atrophy after cardiac surgery, which, to our knowledge, is novel. The patient demonstrated bilateral ophthalmoplegia, with gradual improvement in voluntary smooth pursuit but persistent impairment in saccadic eye movements. Interestingly, the patient showed improved proprioceptive-driven pursuit of their own hand compared to pursuit or saccades following an examiner's hand. The visual field examination revealed a bilateral paracentral temporal hemianopic field defect. The underlying mechanisms of ASOMP and potential chiasmal ischemia in this case remain unknown. Clinicians should be aware of the possibility of ASOMP following cardiac surgery, with potential slow improvement over time.

## Introduction

Acquired supranuclear ocular motor paresis (ASOMP) is a rare complication after cardiac surgery. The classical presentation of ASOMP is a supranuclear limitation of volitional eye movements that may preferentially affect saccades or smooth pursuit. Although microemboli and cerebral ischemia at the thalamo-mesencephalic junction following prolonged intraoperative cardiac bypass time have been proposed as possible risk factors, the precise etiology and pathogenesis of ASOMP following cardiac surgery remain ill-defined. Chiasmal ischemia is also a rare finding after cardiac surgery and may produce bitemporal hemianopsia. We report a case of ASOMP and paracentral bitemporal hemianopsia with optic atrophy following cardiac surgery. To our knowledge, this is the first such case to be reported in the English language ophthalmic literature.

## Case presentation

A 49-year-old male presented with bilateral ophthalmoplegia after cardiac surgery. Past medical history was significant for hypertension, hyperlipidemia, morbid obesity, gastroesophageal reflux disease, and obstructive sleep apnea.

**Figure 1:**
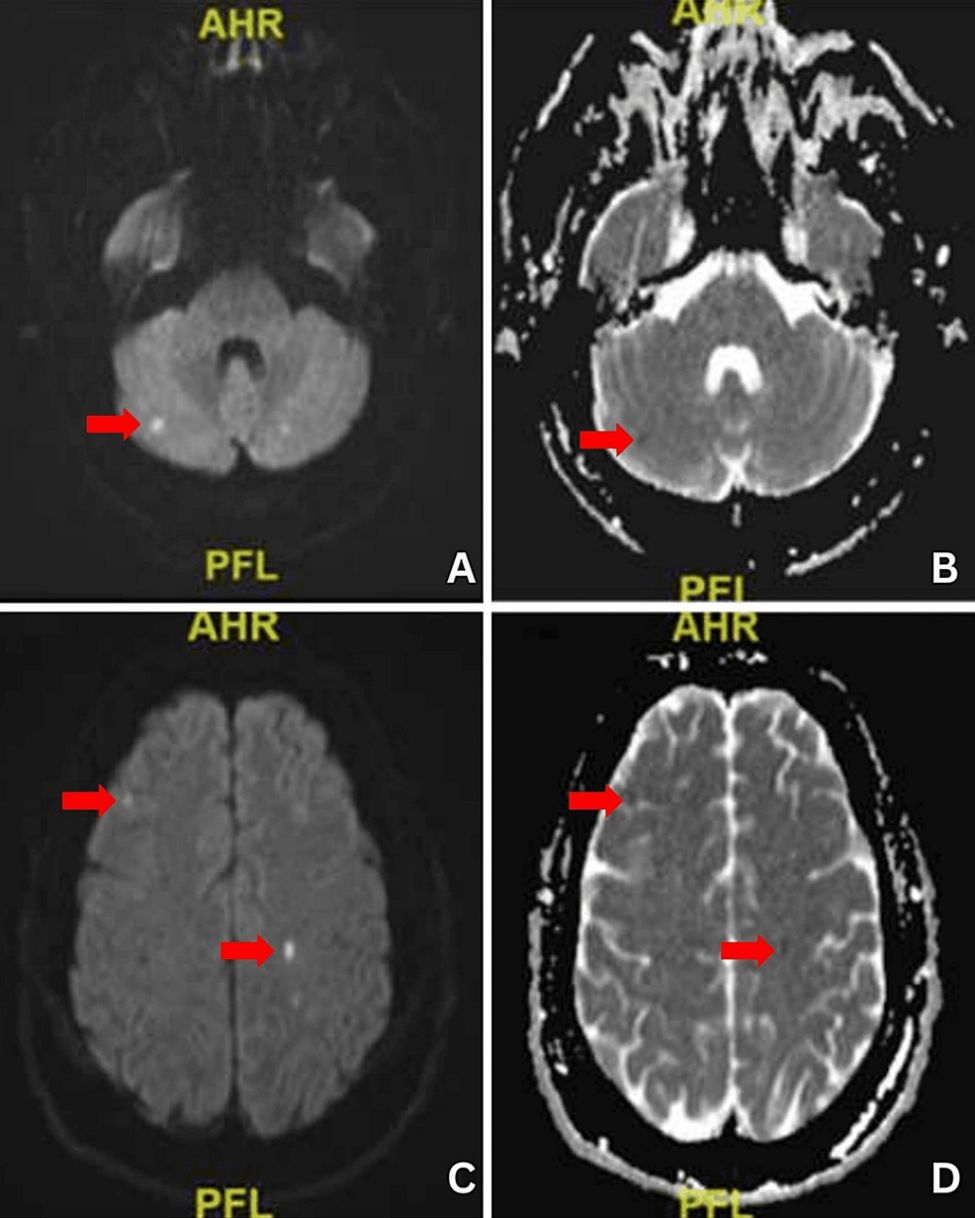
Diffusion-weighted (DWI) axial MRI with arrows demonstrating both supratentorial and infratentorial hyperintense foci that are correlated to hypointense foci in apparent diffusion coefficient (ADC) c/w multiple small acute infarctions

He underwent open heart surgery for acute type A ascending aortic dissection. Postoperatively, he remained intubated, and his hospital course was complicated by a postoperative stroke that presented with left hemineglect, upper extremity numbness, spasticity, and acute renal failure requiring hemodialysis. He was initially anticoagulated with enoxaparin and a transesophageal echocardiogram showed no evidence of cardiogenic emboli. However, magnetic resonance imaging (MRI) of the brain performed one week later showed small vessels and multifocal recent ischemia from emboli (Figure 1).

**Figure 2:**
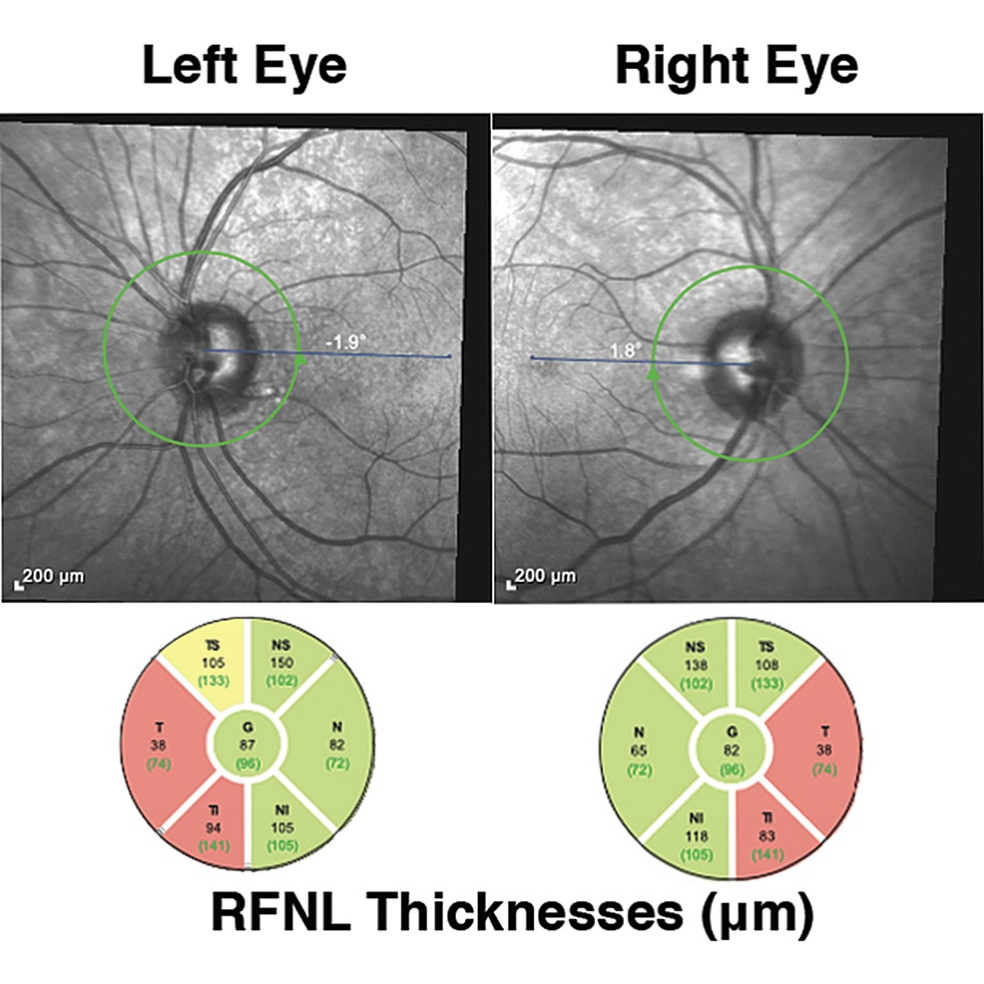
Optical coherence tomography findings suggest global thinning in the nasal fibers corresponding to the bitemporal hemianopsia in Figure 1

He developed a cardiac flutter 13 days after the operation and successfully underwent cardioversion with pacemaker placement. The patient reported that both of his eyes were initially “completely stuck” and “would not move.” On examination, his visual acuity was 20/20 in both eyes (OU). The pupils were isochoric without a relative afferent pupillary defect. There was no ptosis. Slit lamp biomicroscopy, intraocular pressure measurements, and fundoscopy in the hospital were unremarkable OU. On sensory motor exam, the patient demonstrated no movement on attempted up and downgaze to command. The bilateral, symmetric ophthalmoplegia, however, was overcome with the doll’s head maneuver consistent with an acquired supranuclear ocular motor paresis (ASOMP). Elevation of the eye could also be elicited with forced lid closure (Bell’s phenomenon). Serial neuro-ophthalmic examinations demonstrated gradual improvement in his voluntary smooth pursuit but he continued to have a global, bilateral ASOMP for saccadic eye movements. He demonstrated a marked dissociation between saccade and pursuit and, interestingly, was able to follow his own finger (proprioceptive driven pursuit) more easily than following the examiner’s hand as a pursuit or saccadic target (the patient declined to provide images demonstrating the gaze palsy). At his last follow-up automated perimetry (Humphrey visual field 24-2) revealed a mean deviation of -2.04 decibels (dB) in the right eye (OD) and -1.77 dB in the left eye (OS). Taken together, the visual field findings 2 of 5 were suggestive of a bilateral paracentral temporal hemianopic field defect (Figure 2).

**Figure 3:**
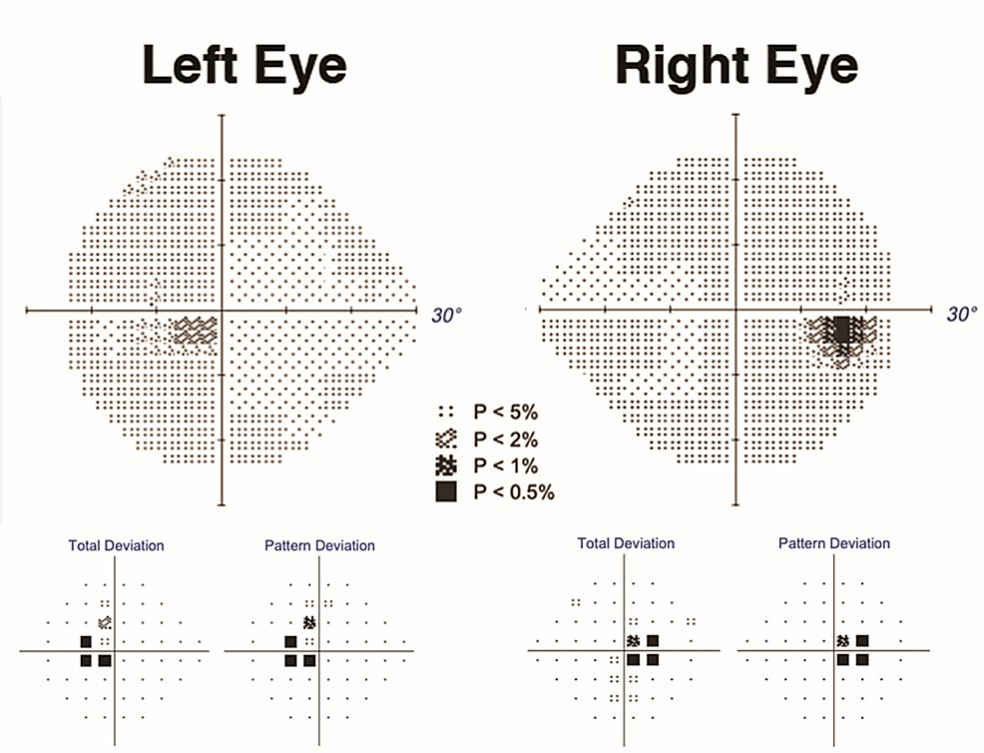
Humphrey 24-2 visual field results show a paracentral bitemporal hemianopia

Ophthalmoscopy and retinal nerve fiber layer (RNFL) optical coherence tomography (OCT) showed thinning of the papillomacular bundles, with global RFNL thicknesses of 87 micrometers (μm) OD and 82 μm OS (Figure 3).

Temporal thickness measured 38 μm bilaterally. However, a repeat brain MRI showed no lesion at the level of the optic chiasm. The patient remained clinically and radiographically stable at the last follow-up four years after cardiac surgery.

## Discussion

Acute onset of ASOMP after cardiac surgery may be associated with global ischemia, microemboli, hypotension, anemia, or prolonged hypothermic cardioplegia [1]. The presumptive localization for ASOMP has been the supranuclear ocular motor control pathways in the thalamo-mesocephalic junction [2], but no definite radiologic correlate on MRI has been found in these cases. Some authors have postulated perineuronal net (PNN) susceptibility to ischemia [3] as the explanation for the lack of imaging correlated to ASOMP. The marked difference between the pursuit eye movements of the patient when following his own hand (proprioceptive-driven pursuit) compared with following the examiner’s hand as a stimulus is fascinating and, to our knowledge, novel in ASOMP. The mechanism of proprioception-driven pursuit is distinct from the known uses of the vestibulo-ocular reflex (VOR) and head thrusting to overcome the ocular motor deficit in cases like congenital ocular motor apraxia. In addition, chiasmal ischemia after cardiac surgery is rare but has been reported [4,5]. The macular nasal crossing fiber decussates in the posterior chiasm. In this case, the paracentral bitemporal hemianopsia had an OCT correlate with papillomacular bundle thinning OU but no radiologic correlate in the posterior chiasm was seen on serial MRI of the sella.

## Conclusions

To our knowledge, the combination of ASOMP and posterior chiasmal ischemia-related bitemporal hemianopsia has not been described previously in the ophthalmic literature. Clinicians should be aware that ASOMP can occur after typical cardiac surgery and that ocular motor deficits may slowly improve with time.
